# G4 & the balanced metric family – a novel approach to solving binary classification problems in medical device validation & verification studies

**DOI:** 10.1186/s13040-024-00402-z

**Published:** 2024-10-23

**Authors:** Andrew Marra

**Affiliations:** Clinical Biostatistician at GE Healthcare, Chicago, IL USA

**Keywords:** G4, P4, MCC, Matthew, Binary classification, MRMC, AUROC, Medical device validation

## Abstract

**Background:**

In medical device validation and verification studies, the area under the receiver operating characteristic curve (AUROC) is often used as a primary endpoint despite multiple reports showing its limitations. Hence, researchers are encouraged to consider alternative metrics as primary endpoints. A new metric called G4 is presented, which is the geometric mean of sensitivity, specificity, the positive predictive value, and the negative predictive value. G4 is part of a balanced metric family which includes the Unified Performance Measure (also known as P4) and the Matthews’ Correlation Coefficient (MCC). The purpose of this manuscript is to unveil the benefits of using G4 together with the balanced metric family when analyzing the overall performance of binary classifiers.

**Results:**

Simulated datasets encompassing different prevalence rates of the minority class were analyzed under a multi-reader-multi-case study design. In addition, data from an independently published study that tested the performance of a unique ultrasound artificial intelligence algorithm in the context of breast cancer detection was also considered. Within each dataset, AUROC was reported alongside the balanced metric family for comparison. When the dataset prevalence and bias of the minority class approached 50%, all three balanced metrics provided equivalent interpretations of an AI’s performance. As the prevalence rate increased / decreased and the data became more imbalanced, AUROC tended to overvalue / undervalue the true classifier performance, while the balanced metric family was resistant to such imbalance. Under certain circumstances where data imbalance was strong (minority-class prevalence < 10%), MCC was preferred for standalone assessments while P4 provided a stronger effect size when evaluating between-groups analyses. G4 acted as a middle ground for maximizing both standalone assessments and between-groups analyses.

**Conclusions:**

Use of AUROC as the primary endpoint in binary classification problems provides misleading results as the dataset becomes more imbalanced. This is explicitly noticed when incorporating AUROC in medical device validation and verification studies. G4, P4, and MCC do not share this limitation and paint a more complete picture of a medical device’s performance in a clinical setting. Therefore, researchers are encouraged to explore the balanced metric family when evaluating binary classification problems.

**Supplementary Information:**

The online version contains supplementary material available at 10.1186/s13040-024-00402-z.

## Introduction

Binary classification problems are a common occurrence in both clinical and non-clinical settings. Whether it involves a doctor determining if a patient has a disease or not, whether an investor should invest money into a certain company, or whether a student will successfully graduate from high school, binary classification is used every day across multiple domains. In the clinical setting, the development of artificial intelligence (AI) opens a large avenue in solving clinical problems such as disease detection through medical devices. Depending on the clinical objective(s), these AI algorithms can even enhance the decision-making processes of human clinicians. For example, in the realm of point-of-care ultrasound, an FDA-approved deep-learning computer-aided detection software was able to guide non-expert users (i.e., registered nurses with no prior experience in conducting echocardiograms) in performing basic examinations of the heart, and these users were able to acquire echocardiograms with similar diagnostic quality as those obtained by expert sonographers with multiple years of formal training [1]. While not intended to replace the expertise of sonographers providing expert imaging, the software was designed to extend echocardiography access in areas where expert sonographers are scarce or unavailable. Although these AI tools can be promising at first glance, to ensure the accuracy of these new technologies is strong, rigorous clinical validation testing must be performed.

### Multi-reader-multi-case study designs

One clinical trial design that is often recommended by the Food and Drug Administration (FDA) to validate a new technology is the multi-reader-multi-case (MRMC) study design [2–4]. The MRMC involves comparing the classification performance of a group of health care professionals (i.e., the readers) using a new technology versus the classification performance of a group of health care professionals using a current standard of care. The two different groups are often labeled as “modalities.” For each modality, the readers will perform testing on a sample of subjects / patients who represent the intended-use population (i.e., the cases). To determine the classification performance of the readers, a reference standard known as the “gold standard” or “ground truth” is first established. The nature of the reference standard varies according to the clinical domain, but one common approach is to use independent expert clinicians with strong domain knowledge who will remain separate from the readers. The ratings of the readers from both modalities will be compared to the same reference panel to produce an overall classification performance score for each reader. After this, the classification performance scores can be compared both among readers and among modalities. Since the variability of each reader’s ability can impact the true performance of the new technology and / or current standard of care, the FDA recommends as many readers as possible to evaluate all cases under each modality. These readers can follow a nested design where they are unique to just one of the modalities, or the same readers can follow a crossed design where they are used in multiple modalities. A fully crossed MRMC design involves the same readers evaluating the same cases across all modalities. Although nested MRMC designs are acceptable for clinical validation and in certain cases more appropriate, the FDA recommends the fully crossed design due to the balanced data structure and increase in statistical power that it provides. Whether a nested or crossed design is utilized, the overall goal of a MRMC study is to reveal any statistically significant differences in classification performance between two or more modalities while also adjusting for the variation that may occur among readers and cases. If this goal is achieved, a reported effect size that shows either superiority or non-inferiority of a new technology when compared to the current standard of care can then be used as justification of the new technology’s clearance for use in a clinical setting [5].

### Defining classification performance in MRMC study designs

The primary metric used to define binary classification performance varies according to the study domain, but in the realm of MRMC study designs, the FDA recommends the area under the receiver operating characteristic curve (AUROC) as the primary endpoint [3, 5]. Before delving into this metric, a researcher needs to be aware of the four main conditional probabilities used in assessing binary classification performance – sensitivity, specificity, the positive predictive value, and the negative predictive value. These four probabilities represent the four possible outcomes that the researcher observes when a classifier is compared to a reference standard. Such outcomes are usually presented in a 2 × 2 contingency table known as a confusion matrix (Table 1). For illustrative purposes, a binary classifier’s scope in this manuscript will be to detect whether a disease is present (i.e., positive) or absent (i.e., negative) in a patient.

1. Sensitivity – given that a patient is truly positive, what is the probability that the new technology will predict the patient as positive? Sensitivity is also known as the True Positive Rate (TPR) or Recall.
2. Specificity – given that a patient is truly negative, what is the probability that the new technology will predict the patient as negative? Specificity is also known as the True Negative Rate (TNR).
3. Positive Predictive Value (PPV) – given that the new technology predicts a patient as positive, what is the probability that the patient is truly positive? PPV is also known as Precision.
4. Negative Predictive Value (NPV) – given that the new technology predicts a patient as negative, what is the probability that the patient is truly negative?

**Table 1:** Confusion matrix for a binary classifier testing the disease status of a patient

	*Patient Is Truly Positive*	*Patient Is Truly Negative*
*Patient Predicted as Positive by New Technology*	**Number of True Positives (TP)**	**Number of False Positives (FP)**
*Patient Predicted as Negative by New Technology*	**Number of False Negatives (FN)**	**Number of True Negatives (TN)**

The mathematical formulas used to calculate the four conditional probabilities are presented below:

$$\begin{array}{c}\text{Sensitivity }=\text{ TP }/ (\text{TP }+\text{ FN})\\ \text{Specificity }=\text{ TN }/ (\text{TN }+\text{ FP})\\ \text{PPV }=\text{ TP }/ (\text{TP }+\text{ FP})\\ \text{NPV }=\text{ TN }/ (\text{TN }+\text{ FN})\end{array}$$

When reporting the four conditional probabilities, two other important statistics are the prevalence and bias of the confusion matrix. These two statistics are defined below:

$$\begin{array}{c}\text{Prevalence }= (\text{TP }+\text{ FN}) / (\text{TP }+\text{ TN }+\text{ FP }+\text{ FN})\\ \text{Bias }= (\text{TP }+\text{ FP}) / (\text{TP }+\text{ TN }+\text{ FP }+\text{ FN})\end{array}$$

When applied to disease detection, prevalence is the proportion of patients in the dataset who are truly positive, and bias is the proportion of patients predicted as positive by the new technology. Mathematically, sensitivity and specificity do not depend on the prevalence of the disease (i.e., prevalence-agnostic), while the positive predictive value and negative predictive value do. Conversely, the positive predictive value and negative predictive value do not depend on the bias of the new technology (i.e., bias-agnostic), while sensitivity and specificity do. When the prevalence and bias are both equal but not equal to 50%, then sensitivity = PPV and specificity = NPV. When the prevalence and bias are both equal to 50%, the confusion matrix becomes fully balanced, which results in all four conditional probabilities being equal.

Usually, a classifier such as an AI will not necessarily report only binary classifications for its predictions. Instead, it may report the predictions as a probability value between zero (i.e., disease is absent) and one (i.e., disease is present), and then a probability threshold will be used to define what is a positive case and what is a negative case. A common threshold is 50%, where probabilities above the threshold are declared as positive predictions, and probabilities below the threshold are declared as negative predictions [6, 7]. A confusion matrix is then produced, and the four conditional probabilities mentioned above can be calculated. Changing the threshold creates a new and often unique confusion matrix, yielding many possible confusion matrices with their own respective conditional probabilities for each threshold. AUROC attempts to aggregate all of these possible confusion matrices by gathering the sensitivity and specificity from each threshold choice. These sensitivity / specificity pairs are plotted on a two-dimensional graph, and the points are then connected by lines to produce what is known as a receiver operating characteristic (ROC) curve. To create a ROC curve, the sensitivity is plotted on the y-axis, and one minus the specificity (also known as the false positive rate) is plotted on the x-axis. Calculating the area under this curve is known as the AUROC, and the area can range between 50 and 100%. An AUROC of 50% is often interpreted as a classifier making a random guess (i.e., a coin flip), while an AUROC of 100% is interpreted as perfect classification performance. There are various methods used to calculate the AUROC, but one approach is the empirical / trapezoidal rule, where the region under the ROC curve is approximated as a collection of trapezoids, and the areas of these trapezoids are calculated and summed together [8]. In essence, AUROC can be viewed as the average sensitivity of a classifier across a range of all possible specificities, and it is often presented as a measure of overall accuracy of the classifier. For a MRMC study design, AUROC is calculated for each reader within each modality, and a reader-average AUROC is also reported. The reader-average AUROC for the new technology modality is statistically compared to the reader-average AUROC of the current standard of care modality, and if the difference in AUROC is statistically significantly greater than zero (with 95% confidence, for example), then there is clinical evidence that the new technology outperforms the current standard of care [2, 3].

### Perils in the misuse of AUROC

The ROC curve has practical use in the medical device validation setting, such as estimating an optimal probability threshold where sensitivity and specificity are the highest, or when initially building a machine-learning model before clinical validation. AUROC is also a well-established measure of a binary classifier’s performance. Due to its focus on optimizing the prevalence-agnostic sensitivity and specificity, AUROC becomes a recommended performance metric when comparing datasets with different prevalence rates. However, despite the added benefit of being prevalence-agnostic, AUROC shares the same limitation that sensitivity and specificity share in that it is not bias-agnostic. Bias often changes as class imbalance in a dataset rises, and a change in overall bias may inflate or deflate the resulting AUROC value leading to unreliable estimates of performance. Therefore, care must be taken when determining whether to use such a measure as a primary endpoint [6, 9, 10].

As mentioned previously, AUROC can be viewed as an average sensitivity across a spectrum of specificity values. In the context of an AI detecting a disease, AUROC is the AI’s ability to rank a truly positive case with a higher predicted probability than a truly negative case. As a result, this only paints a partial picture and ignores the inverse context – if the AI predicts that a disease is present or absent, is the AI truly accurate? In other words, AUROC only accounts for sensitivity and specificity while ignoring PPV and NPV, which are also important for validating disease detection. For example, a MRMC analysis involving a sample of patients who are representative of the true population can reveal that the AI performs slightly better than the current standard of care according to the AUROC of the two modalities, which leads to a researcher reporting no clinically meaningful improvement. But the AI could have also gained a large improvement in PPV and / or NPV, yet this improvement cannot be expressed when using AUROC alone. This is not a weakness of AUROC, but rather a function of its intended use as a means to optimize sensitivity and specificity alone.

One simple solution to this problem is to report all four conditional probabilities in addition to AUROC. But in the setting of MRMC studies where inferential testing is often required, the inclusion of multiple endpoints will also bring about the multiple comparisons problem. If this problem is ignored, then statistical inference will often lead to confidence intervals that are too narrow (i.e., a larger Type I Error rate). Conversely, if the problem is addressed using a statistical technique that adjusts for these multiple inferences (whether it be a direct adjustment to the confidence interval or a gate-keeping approach), the adjustment may lead to confidence intervals that are too wide (i.e., a larger Type II Error rate). Even if the multiple comparisons problem did not exist, the confusion matrix is often not reported in published research involving MRMC study designs, and only AUROC or sensitivity / specificity are reported [5]. This is understandable, since AUROC is the recommended primary endpoint according to the FDA, and incorporating other facets of the confusion matrix may complicate the justification of whether a study objective was satisfied and whether an AI can be cleared for use in a clinical setting. Although the FDA allows other metrics to be considered alongside AUROC, AUROC is still the most popular choice, and researchers and regulatory agencies must realize the limitations of using AUROC as a sole primary endpoint in the clinical testing of medical devices [6].

In certain situations, only using AUROC may be appropriate, and AUROC can still provide a reasonable measure of performance. For example, if the study design depends solely on maximizing sensitivity or specificity and does not consider false positives / negatives as equally harmful, then AUROC is a reasonable choice. Another example is when analyzing a dataset that is fully balanced or approximately balanced. In this case, sensitivity and specificity provide just as much information as PPV and NPV. In the context of clinical validation using a MRMC study design, the above two examples rarely occur. The population prevalence of a disease is often low (< 10%), and dataset imbalance is expected [11–13]. Therefore, accurate disease detection requires strong performance on all four conditional probabilities, not just sensitivity and specificity. Unfortunately, by ignoring PPV / NPV and focusing only on AUROC as a measure of classifier performance, the use of AUROC in this context will lead to statistical biases and a portrayal of the classifier’s true performance as weaker than the truth [6]. Conversely, when the prevalence of a disease is large, AUROC will tend to overestimate the true performance of an AI by placing too much weight on sensitivity alone despite a potentially poorer specificity. This can create misleading conclusions at both the standalone level and when comparing different modalities to each other in the MRMC study. The objective of this manuscript is to present alternative metrics that paint a more complete picture of a classifier’s performance, and compare the performances of such metrics to AUROC. A new metric will also be introduced, called G4, that expresses a classifier’s true potential and can serve as a substitute to AUROC when applied to medical device validation and verification studies.

### G4 & the balanced metric family

G4 is a metric that incorporates all four conditional probabilities of the confusion matrix, and is represented by the formula below:

$$\mathbf{G}4=(\mathbf{T}\mathbf{P}\mathbf{R}\mathbf{*}\mathbf{T}\mathbf{N}\mathbf{R}\mathbf{*}\mathbf{P}\mathbf{P}\mathbf{V}\mathbf{*}\mathbf{N}\mathbf{P}\mathbf{V})\hat{\phantom{0}}(1/4)$$

When expressed in the form of TP, TN, FP, and FN, the formula for G4 becomes:

$$\mathbf{G}4=((\mathbf{T}\mathbf{P}\mathbf{*}\mathbf{T}\mathbf{N})/((\mathbf{T}\mathbf{P}+\mathbf{F}\mathbf{N})\mathbf{*}(\mathbf{T}\mathbf{N}+\mathbf{F}\mathbf{P})))\hat{\phantom{0}}(1/2)\mathbf{*}((\mathbf{T}\mathbf{P}+\mathbf{F}\mathbf{N})\mathbf{*}(\mathbf{T}\mathbf{N}+\mathbf{F}\mathbf{P})/((\mathbf{T}\mathbf{P}+\mathbf{F}\mathbf{P})\mathbf{*}(\mathbf{T}\mathbf{N}+\mathbf{F}\mathbf{N})))\hat{\phantom{0}}(1/4)$$

G4 is the geometric mean of the four conditional probabilities, with a range between 0 and 1. A value close to 0 indicates poor classification performance, and a value close to 1 indicates that all of the conditional probabilities are large and the classification performance is excellent. Depending on a researcher’s goal, this metric can be weighted such that certain conditional probabilities are given greater emphasis than others. The general form of G4 can thus be expressed using the formula below:

$$\mathbf{G}4=(\mathbf{T}\mathbf{P}\mathbf{R}\hat{\phantom{0}}({\varvec{w}}1)\mathbf{*}\mathbf{T}\mathbf{N}\mathbf{R}\hat{\phantom{0}}({\varvec{w}}2)\mathbf{*}\mathbf{P}\mathbf{P}\mathbf{V}\hat{\phantom{0}}({\varvec{w}}3)\mathbf{*}\mathbf{N}\mathbf{P}\mathbf{V}\hat{\phantom{0}}({\varvec{w}}4))\hat{\phantom{0}}(1/{\sum }_{{\varvec{i}}=1}^{4}{\varvec{w}}{\varvec{i}})$$

where *w* is the weight / emphasis given for each conditional probability. For the scope of this manuscript, only G4 with equal weights will be considered.

Although other metrics exist that include a combination of two or more of the conditional probabilities, very few of them include all four. A table of known metrics used in binary classification problems is listed in Table 2.

**Table 2:** Metrics used in binary classification problems [12, 14–18]

Metric Name	Formula
Accuracy	(TP + TN) / (TP + TN + FP + FN)
Misclassification Rate	(FP + FN) / (TP + TN + FP + FN)
Balanced Accuracy	(TPR + TNR) / 2
Geometric Mean	(TPR * TNR) ^ (1/2)
Fowlkes-Mallows Index	(TPR * PPV) ^ (1/2)
F_1_ Score (Sørensen-Dice Coefficient)	2 / (1 / TPR + 1 / PPV)
Jaccard Index (Intersection Over Union)	TP / (TP + FP + FN)
Percent Positive Agreement (F_1_^+^ Score)	(2 * TP) / ((2 * TP) + FP + FN)
Percent Negative Agreement (F_1_^−^ Score)	(2 * TN) / ((2 * TN) + FP + FN)
Unified Performance Measure (P4 Metric)	4 / (1 / TPR + 1 / TNR + 1 / PPV + 1 / NPV)
Youden’s J Statistic (Bookmaker Informedness)	TPR + TNR—1
Markedness	PPV + NPV—1
Cohen’s Kappa	(2 * ((TP * TN)—(FP * FN))) / ((TP + FP) * (FP + TN) + (TP + FN) * (FN + TN))
Matthew’s Correlation Coefficient (MCC)	(TPR * TNR * PPV * NPV) ^ (1/2)—((1—TPR) * (1—TNR) * (1—PPV) * (1—NPV)) ^ (1/2)

AUROC is not listed in Table 2 due to the fact that the above metrics apply only to a single confusion matrix and not towards predicted probabilities. However, balanced accuracy is closely-related to AUROC when evaluating a single confusion matrix. In fact, balanced accuracy is equivalent to AUROC when the predictor is binary and only one possible confusion matrix exists. This will produce only one intermediate sensitivity / specificity point for the ROC curve, and the area under the curve (i.e., AUROC) becomes the arithmetic mean of the sensitivity and specificity [19].

G4 is similar to the Geometric Mean and the Fowlkes-Mallows Index and can be expressed as an extension of the two metrics. Of the metrics listed in Table 2, only the Unified Performance Measure (for the sake of simplicity, the alternate label of P4 will be used in this manuscript) and MCC incorporate all four conditional probabilities. P4 is an extension of the F_1_ Score that incorporates specificity and NPV, while MCC is equivalent to the Pearson Product Correlation Coefficient when applied to two binary variables [14, 17, 18]. Another family of metrics based on the harmonic mean of the conditional probabilities, known as the General Performance Score or GPS, has also been published [15]. In fact, all of these metrics are closely related to G4 and can be considered as part of a balanced metric family. Unfortunately, this family of metrics is rarely considered in research publications, while metrics such as AUROC, Cohen’s Kappa, and balanced accuracy remain popular despite multiple reports that show the limitations of such metrics [6, 10, 20, 21]. The goal of this manuscript is to shed more light on the benefits and intuitive nature of the balanced metric family. To simplify the manuscript’s scope, the balanced metric family will consist of G4, P4, and MCC only, although GPS can still apply since P4 is a special case of GPS.

## Methods

To evaluate the performance of the balanced metric family, a mathematical review will first be conducted. This will help define such metrics and reveal their application to medical device validation and verification studies. Secondly, the metrics will be compared to AUROC using simulated datasets involving difference prevalence rates; appropriate use of the metrics will also be assessed. Benchmark testing will be conducted to assist researchers who wish to use the metrics for inferential analyses. Finally, the reliability of the balanced metric family will be tested on data from an independently published study. All simulations and data analyses were performed using the statistical software R Version 4.2.2 [22]. R code for the simulations and data analyses will be provided as supplementary documentation to this manuscript.

### Mathematical review of the balanced metric family

G4 follows a multiplicative design, while MCC and P4 follow an additive and harmonic design, respectively. For P4, this is immediately noticeable since it is equivalent to the harmonic mean of the four conditional probabilities. For MCC, the formula can be expressed as a linear combination of G4 and the four primary conditional probabilities, where:

$$\text{MCC }= (\text{G}4) \hat{\phantom{0}} 2 - ((1 -\text{ TPR}) * (1 -\text{ TNR}) * (1 -\text{ PPV}) * (1 -\text{ NPV})) \hat{\phantom{0}} (1/2)$$

The relationship among the three balanced metrics is further evident when the prevalence and bias are equal, which results in TPR = PPV and TNR = NPV:

$$\text{G}4 \to (\text{TPR }*\text{ TNR }*\text{ TPR }*\text{ TNR}) \hat{\phantom{0}} (1/4) = (\text{TPR }*\text{ TNR}) \hat{\phantom{0}} (1/2) =\text{ Geometric Mean of Sensitivity and Specificity}$$

$$\text{P}4 \to 4 / (1 /\text{ TPR }+ 1 /\text{ TNR }+ 1 /\text{ TPR }+ 1 /\text{ TNR}) = 4 / ((2 /\text{ TPR}) + (2 /\text{ TNR})) = 2 / ((1 /\text{ TPR }+ 1 /\text{ TNR})) =\text{ Harmonic Mean of Sensitivity and Specificity}$$

$$\text{MCC }\to (\text{G}4) \hat{\phantom{0}} 2 - ((1 -\text{ TPR}) * (1 -\text{ TNR}) * (1 -\text{ TPR}) * (1 -\text{ TNR})) \hat{\phantom{0}} (1/2) =\text{ TPR }*\text{ TNR }- (1 -\text{ TPR}) * (1 -\text{ TNR}) =\text{ TPR }+\text{ TNR }- 1 =\text{ Youden's J Statistic}$$

While the possible values for G4 and P4 are between 0 and 1, the range for MCC is between -1 and 1. To match the range across all three metrics, MCC can be scaled between 0 and 1 using the following formula:

$${\text{MCC}}_{\text{scaled}} = (\text{MCC }+ 1) / 2$$

Expressing the previous MCC equation in the form of MCC_scaled_, the outcome is:

$${\text{MCC}}_{\text{scaled}} = \left(\text{Youden's J Statistic }+ 1\right) / 2 = \left(\text{TPR }+\text{ TNR}\right)/ 2 =\text{ Balanced Accuracy }=\text{ Arithmetic Mean of Sensitivity and Specificity}$$

Based on the above findings, all three balanced metrics can be expressed in the form of a geometric (G4), harmonic (P4), or arithmetic (MCC_scaled_) mean. According to the arithmetic / geometric / harmonic mean inequality, the arithmetic mean ≥ geometric mean ≥ harmonic mean, which corresponds to MCC_scaled_ ≥ G4 ≥ P4. When calculating the difference between two metrics of the same form, the inequality reverses such that P4_Diff_ ≥ G4_Diff_ ≥ MCC_scaled-Diff_. All three metrics are equivalent when both the prevalence and bias are equal to 50% (i.e., a fully balanced dataset), and all three metrics are consistent even if the order of the class labels is reversed (i.e., the positive class is labeled as the negative class and vice versa). Since MCC_scaled_ simplifies to balanced accuracy when a dataset is fully balanced, the entire balanced metric family simplifies to balanced accuracy as well. This finding reveals the relationship between the balanced metric family, balanced accuracy, and to a certain extent, AUROC.

### The balanced metric family Vs. AUROC

Although the balanced metric family is closely related to AUROC when dealing with balanced datasets, as class imbalance increases, the relationship dissolves. The balanced metric family joins two extra probabilities in the form of PPV and NPV to account for any class imbalance, while AUROC does not. By incorporating the prevalence of a dataset into an analysis, an extra layer of precision is added to the true classification performance [6]. Combining all four conditional probabilities into one metric also eliminates the multiple comparisons problem and simplifies an inferential analysis. However, incorporating prevalence is a delicate process, especially when applied to inferential analyses such as those performed in a MRMC study design [3, 23]. Therefore, it is essential to explore the behaviors of the balanced metric family at different magnitudes of prevalence. This can be done through the use of a simulation.

A simulation was created involving 10,000 unique observations, with each observation representing a potential patient in a clinical study. The parameters of the simulation were set up as follows:

1. Begin with assigning an AUROC and a prevalence / bias rate of the minority class for the simulation run. For the first iteration, AUROC = 50% and prevalence = bias = 50%. Although the bias of a binary classifier may potentially be lower or higher than the prevalence of a dataset, it is fair to assume equality as a baseline since the classifier is also assumed to be well-trained and cross-validated prior to clinical validation.
2. Generate 10,000 simulated probabilities such that they produce an AUROC of 50% with a prevalence of 50%. The simulated probabilities can be obtained using the method described by Salgado, J. (2018), where AUROC is converted to Cohen's D and the data is sampled from two standard normal distributions that are D standard deviations apart [24]. The formulas for the conversion process are listed below:
  $$\begin{array}{c}\text{t }= (\text{ ln}(1 / (1 -\text{ AUROC}) \hat{\phantom{0}} 2) ) \hat{\phantom{0}} (1/2)\\ \text{z }=\text{ t }- ( (2.515517 + 0.802853 *\text{ t }+ 0.0103328 *\text{ t }\hat{\phantom{0}} 2) / (1 + 1.432788 *\text{ t }+ 0.189269 *\text{ t }\hat{\phantom{0}} 2 + 0.001308 *\text{ t }\hat{\phantom{0}} 3) )\\ \text{Cohens D }=\text{ z }* 2 \hat{\phantom{0}} (1/2)\end{array}$$
3. Assign a probability threshold of 50% as the binary decision threshold. Simulated probabilities above 50% will be declared as positive predictions, and simulated probabilities less than or equal to 50% will be declared as negative predictions. As mentioned previously, other probability thresholds may be more appropriate for certain study designs, but 50% is used in general and is a practical starting point [6].
4. Create a confusion matrix for the new classification labels built in Step 3, and calculate G4, P4, and MCC_scaled_.
5. Repeat Steps 1 through 4, except that the assigned AUROC is 51% instead of 50%, while maintaining a prevalence / bias of 50%. The assigned AUROC will change sequentially from 50 to 100%, with increments of 1% in between.
6. Repeat Steps 1 through 5, except that the assigned prevalence and bias are 40% instead of 50%. The assignment will change sequentially from 50 to 10%, with increments of 10% in between.

Once the simulation was completed, each value for AUROC had a corresponding value from the balanced metric family across different prevalence rates, and these values were subsequently compared.

### Application of the balanced metric family to an independently published study

The balanced metric family was compared to AUROC using data from an independently published study written by Shen, Y., Shamout, F.E., Oliver, J.R. et al. [13]. This study tested the performance of a unique ultrasound AI in the context of breast cancer detection. According to the authors, breast ultrasound has been noted to produce a large false-positive rate and low precision / PPV. To offset this limitation, a computer-aided diagnosis system using a deep neural network was developed to help radiologists identify and localize malignant lesions in the breast. As an ultrasound exam was conducted, the AI evaluated all input images obtained during the exam and provided a probability of malignancy while also presenting a visual indicator of saliency across each image. As a result, the new technology was able to assist radiologists with interpreting whether a biopsy should be recommended for a patient. To test the performance of the AI, a fully-crossed MRMC study was conducted encompassing 663 exams total and two modalities (i.e., a radiologist using the new technology to detect potential lesions versus the same radiologist not using the technology). Ten board-certified breast radiologists rated each exam according to the Breast Imaging Reporting and Data System, where the rating was a rank score ranging between 1 and 6, with 1 indicating the least potential for malignancy in the breast and 6 indicating the greatest potential. For ratings greater than or equal to 4, the radiologists concluded that there was a strong suspicion for malignancy, and a biopsy was recommended. Scores lower than 4 indicated that a biopsy was not recommended. The objective of the MRMC study was to uncover a statistically significantly greater classification performance for correctly recommending a biopsy when the new technology was utilized (and by extension, for correctly not recommending a biopsy when malignancy is not observed). The results of the study were presented in the form of modality-specific AUROC scores as well as the four conditional probabilities of the confusion matrix. Using these results and the total sample size of the MRMC study, the balanced metric family was subsequently calculated [13].

## Results

### Simulation results & benchmark testing

**Fig. 1:**
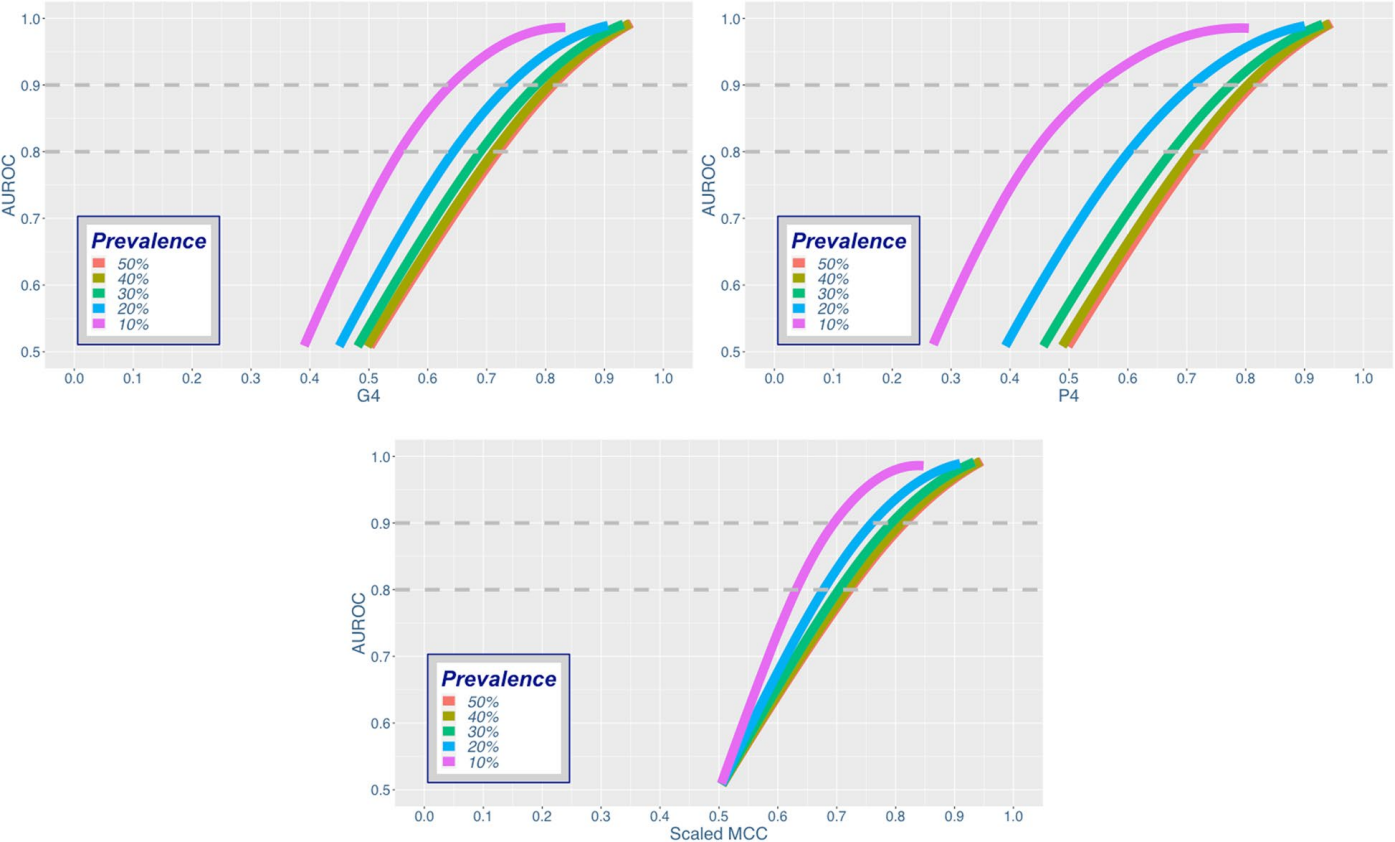
The balanced metric family Vs. AUROC

The simulation results comparing the balanced metric family to AUROC are observed in Fig. 1.

As the prevalence rate increases, the three balanced metrics also increase. When the prevalence equals 50%, the curves for all three metrics are identical, with marginal differences due to sampling error from the simulation process. Since the balanced metric family simplifies to balanced accuracy when the prevalence and bias equal 50%, the respective 50% curves can also be used as a comparison between balanced accuracy and AUROC. Of the three balanced metrics, MCC_scaled_ varied the least across the different prevalence rates (Interquartile Range = 0.176), while P4 varied the most (Interquartile Range = 0.226) and G4 served as a middle ground (Interquartile Range = 0.201). The difference in variation is in line with the difference between arithmetic (MCC_scaled_), geometric (G4), and harmonic (P4) scales. However, when the prevalence was 30% or greater, all three metrics had similar variability, with an Interquartile Range of 0.190, 0.200, and 0.199 for MCC_scaled_, P4, and G4, respectively.

Figure 1 also reveals the intersection where the balanced metric family equals an AUROC of 80% or 90%, two values that are often used as benchmarks for good and excellent classification performance, respectively [25]. The exact values are reported in Table 3.

**Table 3:** Benchmarks for the balanced metric family when compared to AUROC[25]

AUROC	Balanced Metric	Prevalence				
		*10%*	*20%*	*30%*	*40%*	*50%*
80% (Good)	G4	0.549	0.642	0.688	0.711	0.719
	P4	0.438	0.599	0.672	0.706	0.716
	MCC_scaled_	0.630	0.677	0.703	0.717	0.721
90% (Excellent)	G4	0.640	0.739	0.785	0.807	0.813
	P4	0.551	0.711	0.775	0.804	0.811
	MCC_scaled_	0.696	0.760	0.793	0.809	0.814

### Results from the breast cancer study

For the breast cancer study, the reader-average confusion matrices are reported in Table 4. The two confusion matrices represent the new technology modality and the current standard of care modality, respectively. Since the original confusion matrices in the breast cancer study only reported the total sample size (*N* = 663) and four conditional probabilities without the raw counts, the estimated counts reported in Table 4 may be slightly different from the true counts due to rounding error. In addition, since only summary data was available and the raw data itself was unavailable for analysis, another limitation was to assume a nested MRMC study design instead of a fully-crossed design when calculating the balanced metric family. This ignores the potential correlation between modalities but will still account for the variability between cases and readers and still maintain a constant prevalence rate across both modalities. The final results may produce more conservative confidence intervals with wider bounds, although the large sample size and balanced data structure will mitigate this limitation and produce similar conclusions to a fully-crossed-design analysis.

**Table 4:** Confusion matrices for breast cancer MRMC study [13]

	*Breast Cancer Truly Present*	*Breast Cancer Truly Absent*
***New Technology Modality***		
*Breast Cancer Suggested by New Technology*	**45**	**72**
*Breast Cancer Not Suggested by New Technology*	**4**	**542**
***Current Standard of Care Modality***		
*Breast Cancer Suggested by Current Standard*	**44**	**117**
*Breast Cancer Not Suggested by Current Standard*	**5**	**497**

Table 4 reveals that the study involved performance testing on a dataset with a rare disease prevalence. The population prevalence for breast cancer is between 7 and 8% according to Shen, Y., Shamout, F.E., Oliver, J.R. et al. (2021), and the prevalence for Table 4 is 7.4% [13]. Therefore, the study sample is a strong representation of the population of interest. The TPR, TNR, PPV, and NPV for the confusion matrices were 91.8%, 88.0%, 38.0%, and 99.2%, respectively, for the new technology modality, and 90.1%, 80.7%, 27.1%, and 99.1%, respectively, for the current standard of care modality. The results of the MRMC analysis revealed an AUROC of 96.2% (95% Confidence Interval: (94.3%, 97.9%)) for the new technology modality and an AUROC of 92.4% (95% Confidence Interval: (90.5%, 94.4%)) for the current standard of care modality. The difference in AUROC between the two modalities (i.e., AUROC_Diff_) was 3.8% (95% Confidence Interval: (2.8%, 5.2%)). Since AUROC_Diff_ for the breast cancer study was statistically significantly greater than zero, the authors determined there was enough clinical evidence to support the notion that the new technology outperformed the current standard of care [2].

Supplementing the above results, the balanced metric family was calculated for each modality along with the respective 95% confidence intervals. These results are presented in Table 5. The confidence intervals were built using a stratified, clustered, non-parametric bootstrap with case resampling, with each case acting as a cluster and the disease prevalence defined as the stratification variable [26, 27]. The bootstrap is a sampling procedure that is used in inferential analyses where repeated sampling with replacement is performed to mimic the process of taking a unique sample from a population of interest. When the sample is adequately sized, represents the true population, and is free from large statistical outliers, and if the parameter of interest has a finite moment (i.e., if the parameter can be estimated with a mean and variance), the bootstrap provides similar confidence intervals to those produced by parametric procedures such as the Student’s t-Test and Analysis of Variance [28–30].

**Table 5:** Balanced metric family calculations for breast cancer MRMC study

Modality	Metric	Point Estimate (95% Confidence Interval)
New Technology	AUROC	96.2% (94.3%, 97.9%)
	G4	74.6% (70.6%, 78.7%)
	P4	68.7% (63.4%, 74.0%)
	MCC_scaled_	77.5% (74.2%, 80.7%)
Current Standard of Care	AUROC	92.4% (90.5%, 94.4%)
	G4	66.6% (62.6%, 70.4%)
	P4	57.0% (52.1%, 61.9%)
	MCC_scaled_	71.6% (68.5%, 74.5%)
Difference Between Modalities	AUROC	3.8% (2.8%, 5.2%)
	G4	8.0% (2.4%, 13.6%)
	P4	11.6% (4.5%, 18.8%)
	MCC_scaled_	5.9% (1.5%, 10.4%)

In the context of MRMC study designs, the analysis of AUROC is often conducted using a random-effects model where the readers, cases, and their interactions are viewed as random effects, while the modalities are viewed as fixed effects. A clustered dataset violates the assumption of statistical independence, which erroneously leads to smaller standard errors and tighter confidence intervals unless a correction is applied [31–33]. Random-effects models are used to adjust for non-independent data structures, and the cluster bootstrap has been shown to produce confidence intervals that are consistent with those produced by random-effects models when the parametric assumptions were maintained [26]. As an added benefit, since the cluster bootstrap statistic is a non-parametric estimate, it can produce distribution-free confidence intervals that are less biased than those produced by parametric methods when the parametric assumptions are violated [27]. Although there are multiple methods available to statistically analyze data from a MRMC study, each with different advantages, they all share a common goal of producing an unbiased effect size while accounting for the variability of the readers and cases [3, 4, 32, 34].

Using the 10%-prevalence benchmarks listed in Table 3, the new technology reported statistically significantly good and excellent performances according to the balanced metric family. The current standard of care, however, only passed the benchmarks for good performance despite AUROC indicating excellent performance. When considering the difference in performance between the new technology and current standard of care, all metrics revealed a statistically significantly greater difference than zero. However, the point estimates of the balanced metric family were larger than the reported AUROC_Diff_, and for P4 specifically, the lower bound of the 95% confidence interval was larger.

## Discussion

Based on the results reported in Table 3, a researcher will be able to conduct inferential analyses using the balanced metric family. A reasonable rule of thumb to prove good classification performance or higher is to use a benchmark value of 70%, 60%, and 50% when the prevalence is common (> 30%), uncommon (10%—30%), or rare (< 10%). This rule of thumb can be applied to the entire balanced metric family. Even though rules of thumb should not be considered as doctrine, they do provide a researcher with a swift approximation and expectation of how well a classifier is performing before a formal analysis is conducted [25]. By using one of the three balanced metrics and their respective benchmark values, a researcher will be able to incorporate AUROC while also including all four conditional probabilities simultaneously. Depending on a researcher’s objective, using one balanced metric over another may be more favorable. For example, if a researcher expects one of the conditional probabilities to be extremely low compared to the others, P4 is more sensitive to this imbalance due to its harmonic nature and will undervalue the central tendency of the data, while MCC_scaled_ will produce a larger central tendency despite such imbalance [14, 18]. In the context of MRMC study designs, P4 will produce lower standalone estimates of performance than G4 and MCC_scaled_. Conversely, when evaluating the differences between modalities, P4 will produce larger estimates than both G4 and MCC_scaled_. G4 will provide consistent middle-ground results for both a standalone assessment and difference-between-modalities assessment, since a geometric mean is less sensitive to extreme values as opposed to an arithmetic or harmonic mean. To avoid potential statistical biases, researchers are encouraged to report all three metrics or establish a metric in the clinical study protocol before a study begins.

Despite the advantages mentioned above, potential misuse of the balanced metric family is still possible, particularly when comparing datasets with different prevalence rates (for example, comparing a dataset with a prevalence < 10% to a dataset with a prevalence of 50%). AUROC is mathematically agnostic to the prevalence rate, which allows it to be compared to other datasets regardless of the prevalence [3]. Although the balanced metric family is resistant to changes in prevalence when the prevalence is common, the variation of the benchmarks listed in Table 3 begins to grow at lower prevalence rates. Ignoring this can potentially lead to statistical biases. Fully-crossed MRMC study designs involve testing the same cases across all modalities, which ensures that all datasets have the same prevalence rates [2]. Therefore, using the balanced metric family in a fully-crossed MRMC setting is appropriate, while care must be taken with other study designs.

For the confusion matrices of the breast cancer study reported in Table 4, the two classes were based on whether a patient should (or should not) receive a biopsy after an ultrasound exam was completed. Since the decision to recommend a biopsy was founded on a Breast Imaging Reporting and Data System score of 4 or higher, and since the score ranged between 1 and 6, this is equivalent to establishing a probability threshold of 50% if the predictions were probabilities. With this relationship, the benchmarks listed in Table 3 are appropriate measures of performance when conducting inferential analyses on the breast cancer data. AUROC_Diff_ for the breast cancer study was statistically significantly greater than zero, indicating superiority of the new technology over the current standard of care. However, despite the statistically significant results, a reader could surmise that the results are not clinically significant, since both modalities performed exceptionally well with lower confidence bounds of AUROC above 90%. Shen, Y., Shamout, F.E., Oliver, J.R. et al. [13] made a sound decision by reporting all four conditional probabilities alongside AUROC, which revealed a 10.9% improvement in PPV for the new technology [5]. If the confusion matrices were not reported, the improvement in PPV would have been missed since AUROC only considers improvements in sensitivity and specificity [6]. In addition, the balanced metric family revealed a larger gap in performance between the two modalities despite the AUROC of both modalities being similar. This is in line with the improvement in PPV that occurred when using the new technology, and a researcher can clearly observe the superiority of the new technology over the current standard of care as opposed to what AUROC reports. This finding also agrees with published literature reporting the potential overestimation of a classifier’s performance when using AUROC on datasets with increasing class imbalance [6, 10]. If the breast cancer study was only a standalone assessment for the current standard of care, the study would have succeeded due to passing the AUROC benchmark, yet the study would have failed if the balanced metric family was used instead. Therefore, careful and appropriate selection of an endpoint is needed since it can influence the overall study conclusions.

### The balanced metric family’s scope beyond a single confusion matrix

The previous examples pertained to study designs where the binary decision threshold was set at 50%. Although this threshold is commonly used, knowledge of a classifier’s performance across many thresholds delivers a more concrete estimate of its true performance [2, 3]. This is what gives the ROC curve and AUROC their appeal, since they can summarize the change in sensitivity and specificity when the threshold changes, and they can also display the thresholds where sensitivity and specificity are the highest [7]. When multiple thresholds need to be considered, AUROC discriminates a classifier’s performance more easily than a metric calculated from a confusion matrix, even if the metric is a member of the balanced metric family [35]. There are, however, other curves aside from the ROC curve that also display the change in magnitude of specific conditional probabilities across different thresholds. For example, the Precision-Recall curve (PR) follows a similar concept to the ROC curve, except that the recall / sensitivity is plotted on the x-axis and the precision / PPV is plotted on the y-axis. The interpretation of the area under the PR curve (also known as AUPR) is the average PPV across different sensitivities and can be used as an alternate estimate for a classifier’s performance, particularly when a minimum false positive rate is important to the researcher and class imbalance is to be expected [2, 13, 23]. Another curve similar to the ROC curve is the Free Response Operating Characteristic curve (FROC), which substitutes the false positive rate on the x-axis with the number of false positives per subject, cluster, scan, image, etc. FROC is often used when the data has a highly variable number of false positives (such as images collected in a mammography, for example) [2]. More recently, other curve types have also been published such as the Precision-Recall-Gain curve (a calibration of the PR curve that uses a harmonic scale rather than a linear scale to calculate the area under it), equi-PPV and equi-NPV lines (a modified ROC curve that incorporates sensitivity–specificity pairs which meet a specified PPV or NPV criterion onto the ROC space), MCC-F1 curve, and MCC-P4 curve [18, 23, 36, 37]. The interpretations may change as the curve type changes, but all curves share the same three objectives: 1) Determine how well the classifier performs across different probability thresholds, 2) Discover the optimal probability thresholds that maximize the metrics under consideration, and 3) Enhance a classifier’s performance by adjusting for the limitations of the general ROC curve and AUROC.

As mentioned throughout this manuscript, metrics incorporating all four conditional probabilities provide more truthful estimates of a classifier’s performance, and the same applies to any curves and the areas under them. However, while extensive literature is present that discusses the ROC, FROC, or PR curves, aside from the references mentioned above, there are few references that explore curves incorporating all four conditional probabilities. Researchers are encouraged to explore these alternatives, since they can potentially enrich one’s interpretation of data when evaluating binary classification problems. Therefore, it is the author’s desire to explore curves and area metrics that incorporate the balanced metric family in a future submission, which may potentially expand the balanced metric family’s scope beyond an analysis of a single confusion matrix.

## Conclusion

The balanced metric family consisting of G4, P4, and MCC_scaled_ provides a more accurate and truthful estimation of a binary classifier’s performance. When considering the balanced metric family instead of AUROC, the true strength of a new technology is expressed, since AUROC does not capture the improvement in PPV / NPV nor addresses the complexity of analyzing datasets with a rare prevalence [6, 10]. This is evident when evaluating the performance of a medical device in a validation and verification study such as a multi-reader-multi-case study. Since the balanced metric family incorporates all four conditional probabilities of the confusion matrix, it can potentially reveal a greater effect size and greater statistical power while also reducing potential statistical biases caused by class imbalance. MCC_scaled_ tends to provide a larger effect size than G4 and P4 when conducting standalone performance assessments, while P4 tends to provide a larger effect size when evaluating the difference in performance between two modalities. G4, however, will provide an optimal middle-ground estimate when both the standalone assessment and difference-between-modalities assessment are equally important. Due to its design as a geometric mean of the conditional probabilities, G4 is also simple to calculate without requiring sophisticated statistical software or feature scaling. Regardless of the metric preference, the balanced metric family delivers an alternative solution for uncovering a classifier’s true potential, and researchers are encouraged to consider this family when solving binary classification problems.

## Supplementary Information

Supplementary Material 1

## Data Availability

Simulation data, data from the breast cancer study, and R scripts used to generate the above tables / figures are available for download on GitHub, at https://github.com/AMarraCap/G4_Metric. Instructions for the simulation processes are present within each script. In addition, two scripts are freely available for researchers: Binary Classification Benchmark Testing Tool - for any desired value of AUROC and according to any desired prevalence / bias, a researcher may be able to obtain a corresponding value from the metrics listed in Table 2 as well as the balanced metric family. This allows researchers to flexibly set benchmarks for inferential analysis according to their needs and expectations. G4 Power Analysis Tool - a power analysis for the G4 metric is available with built-in instructions. This tool will be helpful for any researcher seeking a minimum sample size and / or desired statistical power when performing inferential analyses with G4.

## Abbreviations

AI: Artificial Intelligence
AUPR: Area Under the Precision-Recall Curve
AUROC: Area Under the Receiver Operating Characteristic Curve
AUROC_Diff_: Difference Between Two AUROC Scores
F1: F_1_ Score
FDA: Food and Drug Administration
FN: False Negative
FP: False Positive
FROC: Free Response Operating Characteristic Curve
G4: G4 Metric
G4_Diff_: Difference Between Two G4 Scores
GPS: General Performance Score
MCC: Matthew’s Correlation Coefficient
MCC_scaled_: Scaled Matthew’s Correlation Coefficient
MCC_scaled-Diff_: Difference Between Two MCC_scaled_ Scores
MRMC: Multi-Reader-Multi-Case
NPV: Negative Predictive Value
P4: Unified Performance Measure / P4 Metric
P4_Diff_: Difference Between Two P4 Scores
PPV: Positive Predictive Value / Precision
PR: Precision-Recall
ROC: Receiver Operating Characteristic Curve
TN: True Negative
TNR: True Negative Rate
TP: True Positive
TPR: True Positive Rate / Recall
W1, W2, W3, W4: Optional Weights For Calculating G4

## References

[1] Narang A, Bae R, Hong H, et al. Utility of a Deep-Learning Algorithm to Guide Novices to Acquire Echocardiograms for Limited Diagnostic Use. JAMA Cardiol. 2021;6(6):624–32. 10.1001/jamacardio.2021.0185. 33599681 10.1001/jamacardio.2021.0185 PMC8204203

[2] Food & Drug Administration, Center for Devices and Radiological Health. September 2022. Clinical Performance Assessment: Considerations for Computer-Assisted Detection Devices Applied to Radiology Images and Radiology Device Data in Premarket Notification (510(k)) Submissions. Guidance for Industry and FDA Staff. https://www.fda.gov/media/77642/download.

[3] Gallas BD, Chan HP, D’Orsi CJ, Dodd LE, Giger ML, Gur D, Krupinski EA, Metz CE, Myers KJ, Obuchowski NA, Sahiner B, Toledano AY, Zuley ML. Evaluating imaging and computer-aided detection and diagnosis devices at the FDA. Acad Radiol. 2012;19(4):463–77. 10.1016/j.acra.2011.12.016. Epub 2012 Feb 3. PMID: 22306064; PMCID: PMC5557046. 22306064 10.1016/j.acra.2011.12.016 PMC5557046

[4] Obuchowski NA, Bullen J. Multireader Diagnostic Accuracy Imaging Studies: Fundamentals of Design and Analysis. Radiology. 2022;303(1):26–34. 10.1148/radiol.211593. Epub 2022 Feb 15 PMID: 35166584. 35166584 10.1148/radiol.211593

[5] Dendumrongsup T, Plumb AA, Halligan S, Fanshawe TR, Altman DG, et al. Multi-Reader Multi-Case Studies Using the Area under the Receiver Operator Characteristic Curve as a Measure of Diagnostic Accuracy: Systematic Review with a Focus on Quality of Data Reporting. PLoS One. 2014;9(12):e116018. 10.1371/journal.pone.0116018. 25541977 10.1371/journal.pone.0116018 PMC4277459

[6] Chicco D, Jurman G. The Matthews correlation coefficient (MCC) should replace the ROC AUC as the standard metric for assessing binary classification. BioData Min. 2023;16(1):4. 10.1186/s13040-023-00322-4.PMID:36800973;PMCID:PMC9938573. 36800973 10.1186/s13040-023-00322-4 PMC9938573

[7] Unal I. Defining an Optimal Cut-Point Value in ROC Analysis: An Alternative Approach. Comput Math Methods Med. 2017;2017:3762651. 10.1155/2017/3762651. Epub 2017 May 31. PMID: 28642804; PMCID: PMC5470053. 28642804 10.1155/2017/3762651 PMC5470053

[8] Cruz-Uribe D, Neugebauer CJ. Sharp error bounds for the trapezoidal rule and Simpson's rule. JIPAM. J Inequalities Pure Appl Math [electronic only] 3.4. 2002;49:22. http://eudml.org/doc/123201.

[9] Chicco D, Jurman G. The advantages of the Matthews correlation coefficient (MCC) over F1 score and accuracy in binary classification evaluation. BMC Genomics. 2020;21(1):6. 10.1186/s12864-019-6413-7.PMID:31898477;PMCID:PMC6941312. 31898477 10.1186/s12864-019-6413-7 PMC6941312

[10] Lobo J, Jiménez-Valverde A, Real R. AUC: A misleading measure of the performance of predictive distribution models. Journal of Global Ecology and Biogeography. 2008;17:145–51.

[11] Chicco DT. quick tips for machine learning in computational biology. BioData Mining. 2017;10:35. 10.1186/s13040-017-0155-3. 29234465 10.1186/s13040-017-0155-3 PMC5721660

[12] Julius Sim, Chris C Wright. The Kappa Statistic in Reliability Studies: Use, Interpretation, and Sample Size Requirements. Phys Ther. 2005;85(3):257–268. 10.1093/ptj/85.3.257. 15733050

[13] Shen Y, Shamout FE, Oliver JR, et al. Artificial intelligence system reduces false-positive findings in the interpretation of breast ultrasound exams. Nat Commun. 2021;12:5645. 10.1038/s41467-021-26023-2. 34561440 10.1038/s41467-021-26023-2 PMC8463596

[14] Ana R. Redondo, Jorge Navarro, Rubén R. Fernández, Isaac Martín de Diego, Javier M. Moguerza, and Juan José Fernández-Muñoz. Unified Performance Measure for Binary Classification Problems. In: Intelligent Data Engineering and Automated Learning – IDEAL 2020. Berlin, Heidelberg: 21st International Conference, Guimaraes, Portugal, November 4–6, 2020, Proceedings, Part II. Springer-Verlag,; 2020. p. 104–12. 10.1007/978-3-030-62365-4_10.

[15] De Diego IM, Redondo AR, Fernández RR, et al. General Performance Score for classification problems. Appl Intell. 2022;52:12049–63. 10.1007/s10489-021-03041-7.

[16] Fowlkes EB, Mallows CL. A Method for Comparing Two Hierarchical Clusterings. J Am Stat Assoc. 1983;78(383):553–69. 10.2307/2288117.

[17] Matthews BW. Comparison of the predicted and observed secondary structure of T4 phage lysozyme. Biochim Biophys Acta. 1975;405(2):442–51. 10.1016/0005-2795(75)90109-9. PMID: 1180967. 1180967 10.1016/0005-2795(75)90109-9

[18] Sitarz M. Extending F1 metric, probabilistic approach. Adv Artif Intell Mach Learn; Res. 2023;3(2):1025–38. 10.48550/arXiv.2210.11997.

[19] Muschelli J. ROC and AUC with a Binary Predictor: a Potentially Misleading Metric. J Classif. 2020;37(3):696–708. 10.1007/s00357-019-09345-1. Epub 2019 Dec 23. PMID: 33250548; PMCID: PMC7695228. 33250548 10.1007/s00357-019-09345-1 PMC7695228

[20] Chicco D, Tötsch N, Jurman G. The Matthews correlation coefficient (MCC) is more reliable than balanced accuracy, bookmaker informedness, and markedness in two-class confusion matrix evaluation. BioData Mining. 2021;14:13. 10.1186/s13040-021-00244-z. 33541410 10.1186/s13040-021-00244-z PMC7863449

[21] Chicco D, Warrens MJ, Jurman G. The Matthews Correlation Coefficient (MCC) is More Informative Than Cohen’s Kappa and Brier Score in Binary Classification Assessment. IEEE Access. 2021;9:78368–81. 10.1109/ACCESS.2021.3084050.

[22] R Core Team. R: A language and environment for statistical computing. R Foundation for Statistical Computing, Vienna, Austria. 2018. URL https://www.R-project.org/.

[23] Peter A. Flach and Meelis Kull. Precision-Recall-Gain curves: PR analysis done right. In Proceedings of the 28th International Conference on Neural Information Processing Systems - Volume 1 (NIPS'15). MIT Press, Cambridge, MA, USA. 2015. p 838–846.

[24] Salgado J. Transforming the Area under the Normal Curve (AUC) into Cohen’s d, Pearson’s r pb, Odds-Ratio, and Natural Log Odds-Ratio: Two Conversion Tables. The European Journal of Psychology Applied to Legal Context. 2018;10:35–47. 10.5093/ejpalc2018a5.

[25] David W. Hosmer Jr., Stanley Lemeshow, Rodney X. Sturdivant. First published. Applied Logistic Regression. Book Series: Wiley Series in Probability and Statistics. John Wiley & Sons, Inc. Print ISBN:9780470582473. Online ISBN:9781118548387. 2013. 10.1002/9781118548387.

[26] Field CA, Welsh AH. Bootstrapping Clustered Data. J R Stat Soc Series B Stat Method. 2007;69(3):369–90. 10.1111/j.1467-9868.2007.00593.x.

[27] Deen M, de Rooij M. ClusterBootstrap: An R package for the analysis of hierarchical data using generalized linear models with the cluster bootstrap. Behav Res. 2020;52:572–90. 10.3758/s13428-019-01252-y. 10.3758/s13428-019-01252-y PMC7148287 31089956

[28] Efron B, Tibshirani RJ. An Introduction to the Bootstrap. 1st ed. Chapman and Hall/CRC; 1994. 10.1201/9780429246593.

[29] Efron B. The Jackknife, the Bootstrap, and Other Resampling Plans. Society for Industrial and Applied Mathematics. CBMS-NSF Regional Conference Series in Applied Mathematics. 1982. SN: 9780898711790. https://books.google.com/books?id=JukZvUd4CAcC.

[30] Efron B. Bootstrap Methods: Another Look at the Jackknife. Ann Stat. 1979;7(1):1–26 http://www.jstor.org/stable/2958830.

[31] Killip S, Mahfoud Z, Pearce K. What is an intracluster correlation coefficient? Crucial concepts for primary care researchers. Ann Fam Med. 2004;2(3):204–8. 10.1370/afm.141. PMID: 15209195; PMCID: PMC1466680. 15209195 10.1370/afm.141 PMC1466680

[32] Obuchowski NA. Nonparametric Analysis of Clustered ROC Curve Data. Biometrics. 1997;53(2):567–78. 10.2307/2533958. 9192452

[33] Rutterford C, Copas A, Eldridge S. Methods for sample size determination in cluster randomized trials. Int J Epidemiol. 2015;44(3):1051–67. 10.1093/ije/dyv113. Epub 2015 Jul 13. PMID: 26174515; PMCID: PMC4521133. 26174515 10.1093/ije/dyv113 PMC4521133

[34] Chen M, Kianifard F, Dhar SK. A bootstrap-based test for establishing noninferiority in clinical trials. J Biopharm Stat. 2006;16(3):357–63. 10.1080/10543400600609478. PMID: 16724490. 16724490 10.1080/10543400600609478

[35] Chongomweru Halimu, Asem Kasem, Shah Newaz SH. Empirical Comparison of Area under ROC curve (AUC) and Matthew Correlation Coefficient (MCC) for Evaluating Machine Learning Algorithms on Imbalanced Datasets for Binary Classification. In: Proceedings of the 3rd International Conference on Machine Learning and Soft Computing (ICMLSC ’19). New York: Association for Computing Machinery; 2019. p. 1–6. 10.1145/3310986.3311023.

[36] Cao C, Chicco D, Hoffman MM. The MCC-F1 curve: a performance evaluation technique for binary classification. ArXiv:2006.11278. 2020. 10.48550/arXiv.2006.11278.

[37] Thomas G, Kenny LC, Baker PN, et al. A novel method for interrogating receiver operating characteristic curves for assessing prognostic tests. Diagn Progn Res. 2017;1:17. 10.1186/s41512-017-0017-y. 31093546 10.1186/s41512-017-0017-y PMC6460848

