# Supplementary figures and images for: G4 & the balanced metric family – a novel approach to solving binary classification problems in medical device validation & verification studies
Source: BioData Min. 2024 Oct 23;17:43. doi: 10.1186/s13040-024-00402-z (PMC11515465; doi:10.1186/s13040-024-00402-z)

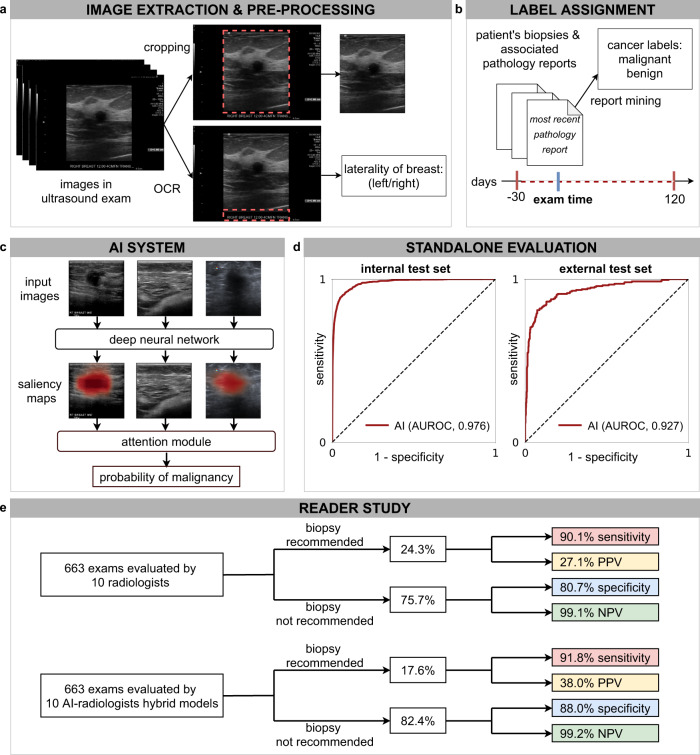

Supplement: Supplementary file 1 — Supplementary Material 1 [file 13040_2024_402_MOESM1_ESM.zip › Supplementary Documents - Data Availability/Breast Cancer AI Study - Reader Study Results.jpg]
